# Anti-PLA2R antibody positivity in adult minimal change disease with tuberculosis co-infection: A case report

**DOI:** 10.1097/MD.0000000000047856

**Published:** 2026-02-20

**Authors:** Xiaokang Chen, Nanxin Li, Qing Yang, Hurui Ling, Heng Yin

**Affiliations:** a Department of Nephrology, Mianyang Central Hospital, School of Medicine, University of Electronic Science and Technology of China, Mianyang, Sichuan, China; b Department of Cardiology, Mianyang Central Hospital, School of Medicine, University of Electronic Science and Technology of China, Mianyang, Sichuan, China; c Department of Clinical Medicine, Chengdu Medical College, Chengdu, Sichuan, China; d Department of Infectious Diseases, Mianyang Central Hospital, School of Medicine, University of Electronic Science and Technology of China, Mianyang, Sichuan, China; e Department of Laboratory Medicine, Mianyang Central Hospital, School of Medicine, University of Electronic Science and Technology of China, Mianyang, Sichuan, China.

**Keywords:** anti-phospholipase A2 receptor (PLA2R), minimal change disease (MCD), pulmonary tuberculosis

## Abstract

**Rationale:**

Anti-phospholipase A2 receptor (PLA2R) antibody is a highly specific serological marker for primary membranous nephropathy, with a detection rate of <2% in minimal change disease (MCD). This case represents the first report of PLA2R-positive adult MCD complicated by active pulmonary tuberculosis (TB) and systemic osteoarthropathy, highlighting the potential for chronic infection to induce PLA2R antibodies through inflammatory activation or molecular mimicry.

**Patient concerns:**

A 73-year-old male with 7-year steroid-dependent nephrotic syndrome developed pulmonary TB and systemic osteoarthropathy during immunosuppressive therapy.

**Diagnoses:**

Serial renal biopsies confirmed MCD/focal segmental glomerulosclerosis (FSGS) without membranous nephropathy features. PLA2R antibody was transiently positive (16.6 RU/mL) during active TB but turned negative after anti-TB treatment. TB was confirmed by next-generation sequencing.

**Interventions:**

A multidisciplinary strategy was implemented: rifapentine replaced rifampicin to maintain tacrolimus levels; rituximab was administered for refractory proteinuria; and TB was treated with a 9-month regimen. Osteoarthropathy was managed with Tripterygium glycosides and joint injections.

**Outcomes:**

Following rituximab administration, 24-hour urinary protein decreased from 6.32 g to 1.5 g. TB was controlled with sputum conversion within 2 months. PLA2R antibody became negative post anti-TB therapy, supporting the hypothesis of infection-induced seropositivity. No renal function decline or TB dissemination occurred.

**Lessons:**

This case suggests that chronic infections like TB may transiently induce PLA2R antibodies via inflammatory activation, highlighting the need for dynamic serologic and histopathologic correlation to avoid misdiagnosis in PLA2R-positive non-MN patients. Multidisciplinary management is critical to balance immunosuppression and infection control.

## 1. Introduction

Minimal change disease (MCD), characterized by diffuse foot process effacement, is a common cause of nephrotic syndrome (NS) in adults.^[1]^ However, its association with anti-phospholipase A2 receptor (PLA2R) antibodies remains controversial. PLA2R antibodies, a serological hallmark of primary membranous nephropathy (MN), are positive in >70% of primary MN patients but rarely detected in MCD (<2%).^[2]^ This discrepancy may stem from overlapping pathologies (e.g., secondary MN) or serological false positivity. Recent studies suggest that chronic inflammatory states (e.g., tuberculosis [TB] infection or autoimmune diseases) may transiently induce PLA2R antibody production via Toll-like receptor (TLR)-mediated polyclonal B-cell activation or molecular mimicry,^[3]^ challenging its traditional role as an MN-specific biomarker.

NS patients face elevated infection risks due to prolonged immunosuppression and hypoalbuminemia, with TB posing a significant threat to chronic kidney disease populations. Epidemiological data indicate a 4 to 30 folds higher TB incidence in chronic kidney disease patients, particularly those on long-term calcineurin inhibitors.^[4]^ However, reports of MCD with active TB are scarce, and the role of PLA2R antibodies in such patients remains undefined. Previous studies focused on bacterial or viral infections (e.g., Streptococcus pneumoniae or hepatitis B virus) in glomerulopathies,^[5]^ while mechanisms linking TB infection to MCD/ FSGS (focal segmental glomerulosclerosis), and clinical management strategies remain unexplored.

This study presents the first case of PLA2R-positive MCD complicated by secondary TB and systemic osteoarthropathy. Key challenges include: 1) interpreting PLA2R positivity in non-MN glomerulopathy, distinguishing chronic inflammation-induced false positivity from early-stage MN; 2) balancing immunosuppression with anti-TB drug interactions (e.g., rifapentine’s impact on tacrolimus metabolism); 3) multidisciplinary management strategies. Through dynamic histopathological monitoring, tailored immunosuppression, and optimized anti-TB therapy, this case offers new perspectives on diagnosing PLA2R-positive non-MN patients and managing high-risk populations.

## 2. Case summary

**Figure 1.**
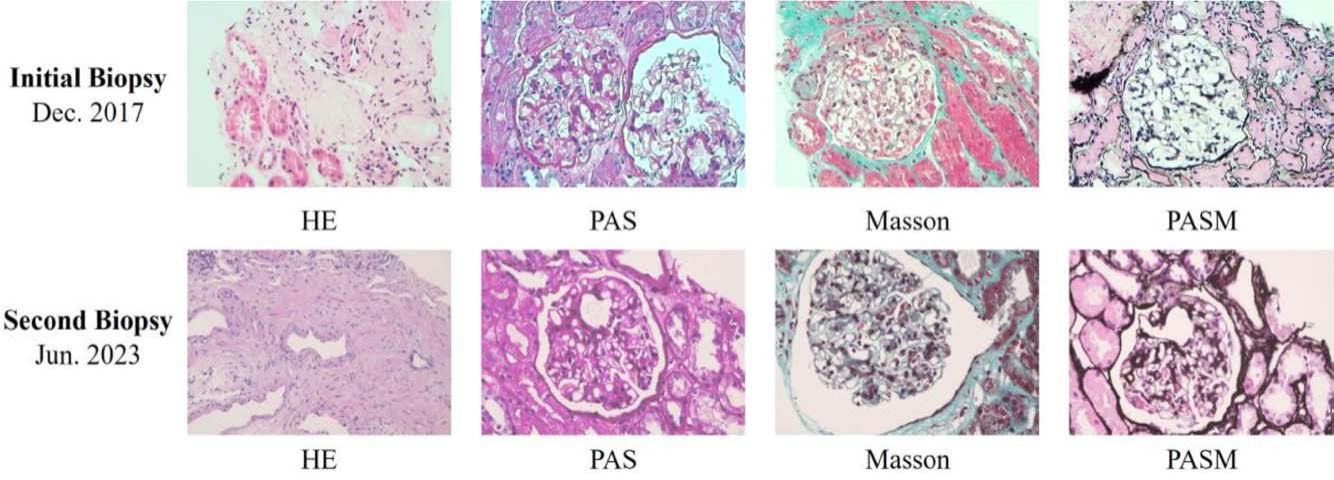
Two renal histopathological examinations. Renal biopsy specimens were routinely stained with HE, PAS, PASM, Masson, Immunofluorescence and electron microscopy. Two renal biopsy reports both indicated MCD or potential FSGS.

A 73-year-old male with a history of grade 2 hypertension (high-risk), hypothyroidism, and gout presented in 2017 with complaints of abdominal distension and generalized edema. Laboratory findings revealed 24-hour urine protein of 5.1 g, plasma albumin of 28.6 g/L, and total cholesterol of 7.62 mmol/L. Renal biopsy demonstrated findings suggestive of MCD or early-stage focal segmental glomerulosclerosis (FSGS) on light microscopy (Fig. 1 and Fig. S1, Supplemental Digital Content, https://links.lww.com/MD/R471), and widespread foot process effacement on electron microscopy (Fig. S2, Supplemental Digital Content, https://links.lww.com/MD/R471). A diagnosis of primary podocytopathy was established.^[6]^ Initial treatment with corticosteroids (methylprednisolone 40 mg/d) combined with ARB therapy resulted in partial remission (urine protein reduction > 90%).

Between 2019 and 2023, the patient experienced multiple relapses. Immunosuppressive therapy was escalated sequentially to cyclosporine (75 mg twice daily) followed by tacrolimus (2 mg twice daily). However, dose adjustments due to drug resistance yielded only transient remission, indicating steroid dependence and CNI resistance. In 2022, the patient developed secondary pulmonary TB (confirmed by next-generation sequencing of alveolar lavage fluid (Table S1, Supplemental Digital Content, https://links.lww.com/MD/R471), which identified *Mycobacterium tuberculosis*). Antituberculosis therapy with rifapentine/isoniazid was administered concurrently with tacrolimus to maintain immunosuppression. In 2023, systemic osteoarthritis developed and was managed with tacrolimus, Tripterygium glycosides, and localized joint injections.

**Figure 2.**
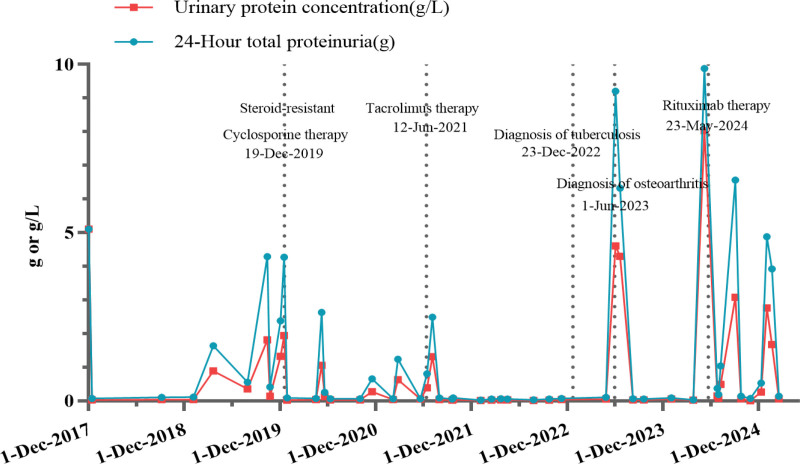
The trend of changes in 24-hour urine protein throughout the course of the disease. December 1, 2017: initial treatment with corticosteroids (methylprednisolone 40 mg/d) combined with ARB therapy resulted in partial remission (urine protein reduction > 90%); December 19, 2019: the patient developed steroid-resistant and received steroid hormone combined with cyclosporine therapy (Prednisone acetate tablets 30mg qd; Cyclosporine 75mg bid), significantly reduced urinary protein; June 12, 2021: the effect of cyclosporine therapy on urinary protein control gradually weakened, and when adjusted to tacrolimus therapy (Tacrolimus: 2mg qd.am, 1.5mg qn), urinary protein was significantly reduced; December 23, 2022: mycobacterium tuberculosis was detected in the bronchoalveolar lavage fluid mNGS, diagnosed as secondary pulmonary tuberculosis, and treated with rifampicin, isoniazid, ethambutol, and pyrazinamide; June 1, 2023: The patient experienced a relapse of nephrotic syndrome due to the onset of osteoarthritis. After controlling the osteoarthritis, tacrolimus combined with Tripterygium glycosides (Tacrolimus 2mg bid; Tripterygium glycosides 20mg bid) maintenance therapy was administered; May 23, 2024: The patient experienced severe recurrence of nephrotic syndrome (24-hour urine protein 6.32 g, serum albumin 22.6 g/L). Following administration of rituximab (RTX, 1.0 g), urine protein decreased to 1.5 g/24 h.

A repeat renal biopsy in 2023 demonstrated mild glomerular lesions with isolated global sclerosis and mild chronic tubulointerstitial damage (Fig. 1 and Fig. S1, Supplemental Digital Content, https://links.lww.com/MD/R471), alongside positive anti-PLA2R antibodies (16.6 RU/mL), raising suspicion of secondary MN. However, at the end of antituberculosis treatment 2 months later, the reexamination of anti-PLA2R antibodies turned negative. In 2024, the patient experienced a severe relapse of NS (24-hour urine protein 6.32 g, serum albumin 22.6 g/L). Following administration of rituximab (RTX, 1.0 g), urine protein decreased to 1.5 g/24 hours. Detailed treatment regimens (Table S2, Supplemental Digital Content, https://links.lww.com/MD/R471) and 24-hour urine protein levels (Fig. 2) are provided below. The specific changes of T-cell/B-cell counts, BUN, Scr, and albumin results of patient at different treatment stages are shown in Figs. S3 and S4 (Supplemental Digital Content, https://links.lww.com/MD/R471).

## 3. Discussion

This is the first report of adult MCD/FSGS with secondary TB and PLA2R antibody positivity. While prior studies emphasize bacterial infections (e.g., *S. pneumoniae*), TB-associated MCD/FSGS is exceptionally rare. This case highlights unique pathology-infection interactions, confirmed by TB diagnosis via next-generation sequencing and exclusion of anti-TB drug-induced MN.

### 3.1. Mechanisms underlying PLA2R antibody positivity

PLA2R positivity without MN pathology may involve: 1) Limitations of ELISA: low-titer antibodies (<20 RU/mL) may yield false positives due to rheumatoid factor or low-affinity antibodies.^[2,3]^ 2) Chronic inflammation-induced B-cell activation: TB infection activates B cells via TLRs, triggering nonspecific antibody production.^[4]^ Recent evidence suggests molecular mimicry between TB antigen Hsp65 and PLA2R epitopes.^[7]^ Interestingly, the anti-PLA2R antibody turned negative at the end of antituberculosis treatment. This provides temporal logical supporting evidence for the hypothetical mechanism that “TB infection induces PLA2R antibodies.” 3) Podocyte injury: Prolonged injury (7-year history, FSGS progression) may expose cryptic PLA2R antigens, inducing autoimmunity.^[2]^ However, absence of MN features (e.g., subepithelial deposits) argues against MN transformation, warranting long-term follow-up. The 3 hypothetical mechanisms are speculative, especially TB infection and PLA2R induction still need to be further verified.

While PLA2R antibody is highly specific, low-titer PLA2R positivity (16.6 RU/mL) should be interpreted with more caution. After reporting positive for PLA2R antibody in this case, we performed another kidney biopsy to rule out PLA2R-positive MN. However, the titer of anti-PLA2R antibody was not significant, which may lead to false positive results or low antibody production, resulting in insufficient renal deposition. This can be distinguished by detecting serum PLA2R antibody subtypes and renal tissue PLA2R immunofluorescence staining. Regrettably, due to the unavailability of serum PLA2R subtype detection in our center and the limited preserved renal tissue samples, we cannot further verify it.

### 3.2. Balancing immunosuppression and TB management

Key to success was reconciling drug metabolism conflicts: Tacrolimus metabolism is primarily dependent on the CYP3A4 enzyme system. Certain components of TB medications, including rifampicin, isoniazid, pyrazinamide, and ethambutol, may modulate the activity of CYP3A4, thereby affecting tacrolimus concentrations. Rifampin, due to its strong CYP3A4 induction effect, can reduce tacrolimus trough concentrations by up to 70%, making dose adjustment of tacrolimus throughout the treatment period extremely challenging. Following the recommendation of the infectious disease department, the treatment was switched to rifapentine (which has a weaker CYP3A4 induction effect). This change significantly reduced the difficulty of maintaining tacrolimus concentrations within the effective therapeutic range (6–8 ng/mL).^[4,6]^ This strategy prevented NS relapse and ensured TB control (sputum conversion within 2 months). Regular liver function monitoring (ALT peaked at 78 U/L) and hepatoprotective therapy aligned with guidelines. In addition, Multidisciplinary collaboration mitigated infection risks post-B-cell depletion (RTX): latent TB screening (T-SPOT negative) and immunoglobulin supplementation (IgG 6.2 g/L) prevented TB reactivation. RTX achieved sustained remission (>6 months), outperforming calcineurin inhibitors, but requires rigorous infection screening and post-treatment monitoring.

### 3.3. Multidisciplinary collaboration

Success relied on nephrology, infectious diseases, and rheumatology teams. Nephrology adjusted immunosuppression based on PLA2R dynamics (16.6 RU/mL → negative) and histopathology (MCD→FSGS), transitioning to RTX. Infectious diseases optimized anti-TB therapy (rifapentine, 9-month regimen). Rheumatology managed osteoarthropathy with tripterygium and intra-articular injections, avoiding NSAID-induced renal injury.

This approach resolved drug interactions, prevented complications (no renal function decline or TB dissemination), and reduced hospitalization duration/costs, offering a replicable model for complex cases.

### 3.4. Study limitations

This study represents the first reported case of anti-PLA2R antibody-positive MCD co-occurring with active TB. The generalizability of our findings is inherently limited by its single-center design, small sample size (n = 1), and the patient’s complex comorbidities (advanced age, hypertension, gout). The proposed mechanistic hypothesis linking TB infection to PLA2R antibody induction – TLR activation or molecular mimicry – lacks direct experimental validation, such as assessment of antigen cross-reactivity or B-cell clonality. Furthermore, repeated renal biopsies lacked PLA2R immunohistochemistry and IgG subclass analysis restricts mechanistic interpretation. Competitive inhibition by other podocyte antibodies (e.g., anti-nephrin) remains speculative and merits targeted serology.

## 4. Conclusions

This is the first report of PLA2R positive MCD with active TB, emphasizing that chronic inflammation may temporarily induce PLA2R antibodies via TLR activation or molecular simulation, but a larger cohort is needed to verify the causal relationship of PLA2R-TB. In PLA2R-positive non-MN cases, dynamic PLA2R monitoring, repeated biopsies, and multidisciplinary collaboration are crucial to avoid misdiagnosis.

## Author contributions

**Conceptualization:** Xiaokang Chen, Heng Yin.

**Investigation:** Xiaokang Chen, Nanxin Li, Qing Yang, Hurui Ling, Heng Yin.

**Methodology:** Xiaokang Chen, Nanxin Li, Qing Yang, Hurui Ling, Heng Yin.

**Validation:** Nanxin Li.

**Visualization:** Heng Yin.

**Writing – original draft:** Xiaokang Chen, Nanxin Li.

**Writing – review & editing:** Xiaokang Chen, Nanxin Li, Heng Yin.

## Supplementary Material
