# Supplementary material for: Anti-PLA2R antibody positivity in adult minimal change disease with tuberculosis co-infection: A case report
Source: Medicine (Baltimore). 2026 Feb 20;105(8):e47856. doi: 10.1097/MD.0000000000047856 (PMC12928962; doi:10.1097/MD.0000000000047856)
Supplement: Supplementary file 1 [file medi-105-e47856-s001.docx]

**Table S1. Results of mNGS.**

| **Species** | **Species name** | **Sequence number** | **（Copies/mL）** |
| --- | --- | --- | --- |
| Bacterium | Haemophilus parainfluenzae | 101 | 450 |
|  | Eikenella corroden | 51 | 224 |
|  | Veillonella parvula | 6 | 100 |
|  | Rothia mucilaginosa | 10 | 124 |
| Virus | - | - | - |
| Fungus | - | - | - |
| **Mycobacteria** | **Mycobacterium tuberculosis** | **615** | **2698** |
| Special pathogen | mycoplasma | - | - |
|  | chlamydia | - | - |
|  | treponemata | - | - |
|  | Rickettsia | - | - |
| Parasite | - | - | - |

**Table S2. Drug treatment schedule.**

| 2017-11-30 | 2019-4-27 | 2019-12-19 |
| --- | --- | --- |
| **Inpatient:**  Methylprednisolone sodium succinate  40mg qd.  **Discharge:**  Prednisone acetate tablets 55mg qd (Tapering the dose in a controlled manner after 8 weeks). | **Inpatient and Discharge:** **Relapse**  Prednisone acetate tablets  10mg 50mg qd (Tapering the dose in a controlled manner after a full treatment course). | **Inpatient:**  Methylprednisolone sodium succinate 40mg qd.  **Discharge:**  Prednisone acetate tablets 30mg qd; Cyclosporine 75mg bid. |
| 2020-05-07 | 2021-06-12 | 2023-04-13 |
| **Inpatient:**  Prednisone acetate tablets 30mg qd; Cyclosporine 50mg bid.  **Discharge:**  Cyclosporine 50mg bid. | **Inpatient:**  Prednisone acetate tablets 30mg qd; Tacrolimus: 2mg qd.am, 1.5mg qn.  **Discharge:**  Tacrolimus: 2mg qd.am, 1.5mg qn. | **Inpatient:**  Tacrolimus: 2mg qd.am, 1.5mg qn; Tripterygium glycosides 20mg bid.  **Discharge:**  Tacrolimus 1.5mg bid; Tripterygium glycosides 20mg bid. |
| 2023-05-20 | 2023-06-01 | 2023-06-19 |
| **Inpatient:**  Tacrolimus: 2mg qd.am, 1.5mg qn; Tripterygium glycosides 20mg bid.  **Discharge:**  Tacrolimus: 2mg qd.am, 1.5mg qn; Tripterygium glycosides 20mg bid. | **Inpatient:**  Methylprednisolone sodium succinate 40mg qd;  Tacrolimus 2mg bid; Tripterygium glycosides 20mg bid.  **Discharge:**  Tacrolimus 2mg bid; Tripterygium glycosides 20mg bid. | **Inpatient:**  Methylprednisolone sodium succinate 40mg qd.  **Discharge:**  Prednisone acetate tablets 30mg qd. |
| 2024-06-05 | 2024-09-04 | 2025-01-09 |
| **Inpatient:**  2024-05-10 Cyclophosphamide 0.2g qod;  2024-05-23/27 Rituximab 500mg.  **Discharge:**  Prednisone acetate tablets 30mg qd. | **Inpatient:**  Rituximab 1g.  **Discharge:**  Prednisone acetate tablets 25mg qd. | **Inpatient:**  Rituximab 1g. |


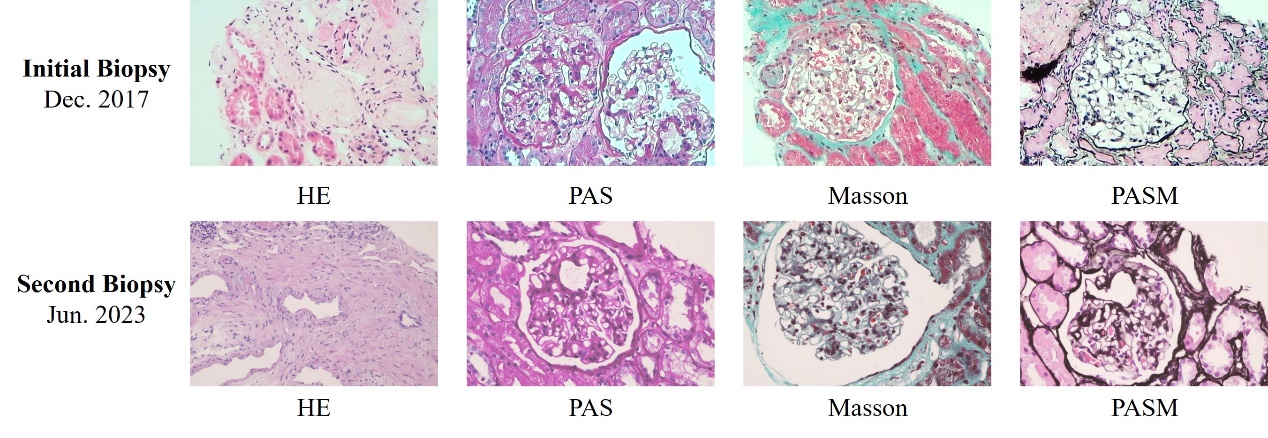


**Figure S1. Two renal histopathological examinations.** Renal biopsy specimens were routinely stained with HE, PAS, PASM, and Masson. Initial biopsy report (Dec. 2017): Light microscopy: 11 glomeruli were observed, with 1 showing global sclerosis. Mild mesangial cell and matrix proliferation were present. The glomerular basement membrane showed no significant thickening, and no spikes, double contours, or eosinophilic deposits were observed. Tubular epithelial cells exhibited granular and vacuolar degeneration, with occasional dilated tubules and loss of brush borders. Scattered interstitial inflammatory cell infiltration was present, but interstitial fibrosis was not significant. Arteriolar walls were thickened, with hyalinosis and luminal narrowing. Immunofluorescence: 5 glomeruli were examined. IgG, IgA, C3, and C1q were negative, while IgM was positive. Second biopsy report (Jun. 2023): Light microscopy: 8-9 glomeruli were observed, with 1 showing global sclerosis. Mild segmental mesangial cell and matrix proliferation were noted. Segmental vacuolar degeneration of the glomerular basement membrane was present. Capillary lumen changes were not significant. Approximately 5% of tubules showed atrophy, with moderate degeneration of tubular epithelial cells. Approximately 5% interstitial fibrosis was observed, accompanied by infiltration of lymphocytes, monocytes, and plasma cells. Arteriolar walls were mildly thickened. Immunofluorescence: 8-10 glomeruli were examined. IgA, κ, λ, IgM, C3, IgG, C4, and C1q were all negative.


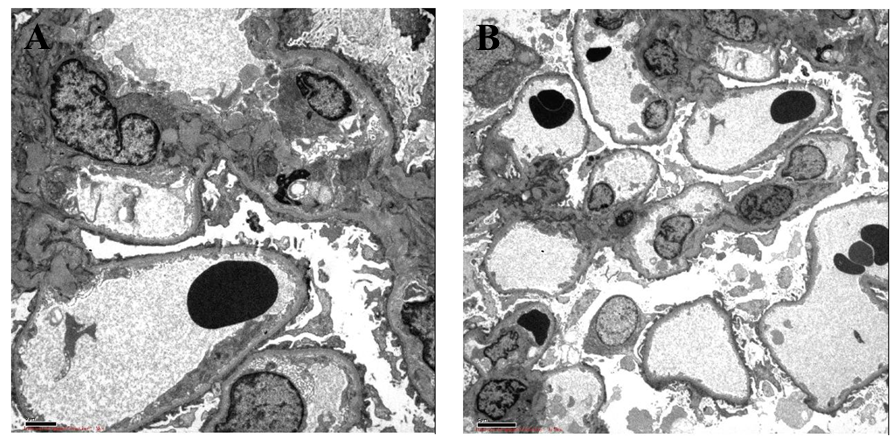


**Figure S2. Electron microscope images of two renal histopathological examinations.** Microscopy of the initial renal biopsy (A) showed swelling of epithelial cells, vacuolar degeneration, diffuse fusion of foot processes, and partial microvilli changes, compliant with podocytosis (MCD or potential FSGS). The second electron microscopy of renal tissue (B) showed that glomerular lesions were mild with individual globulin sclerosis, and widespread foot joint fusion was seen.


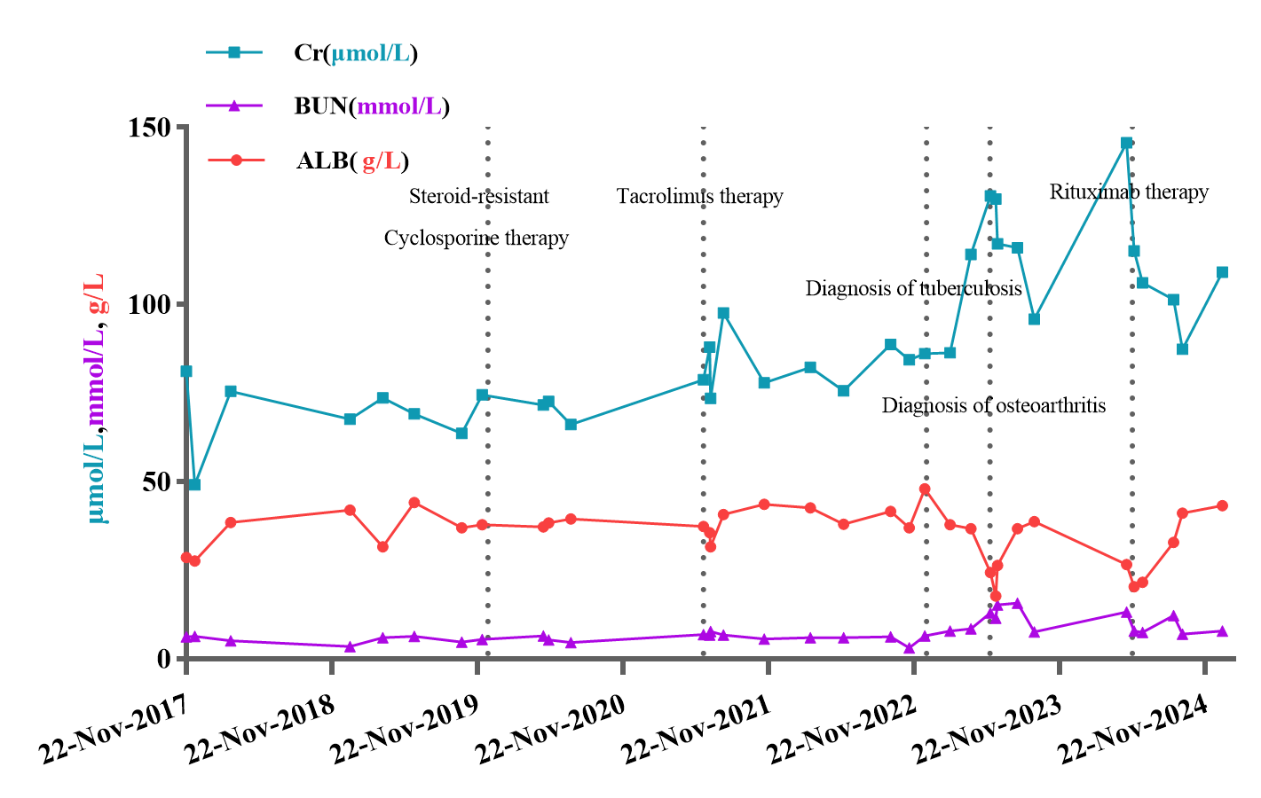


**Figure S3. The trend of changes in BUN, Scr and ALB throughout the course of the disease.**


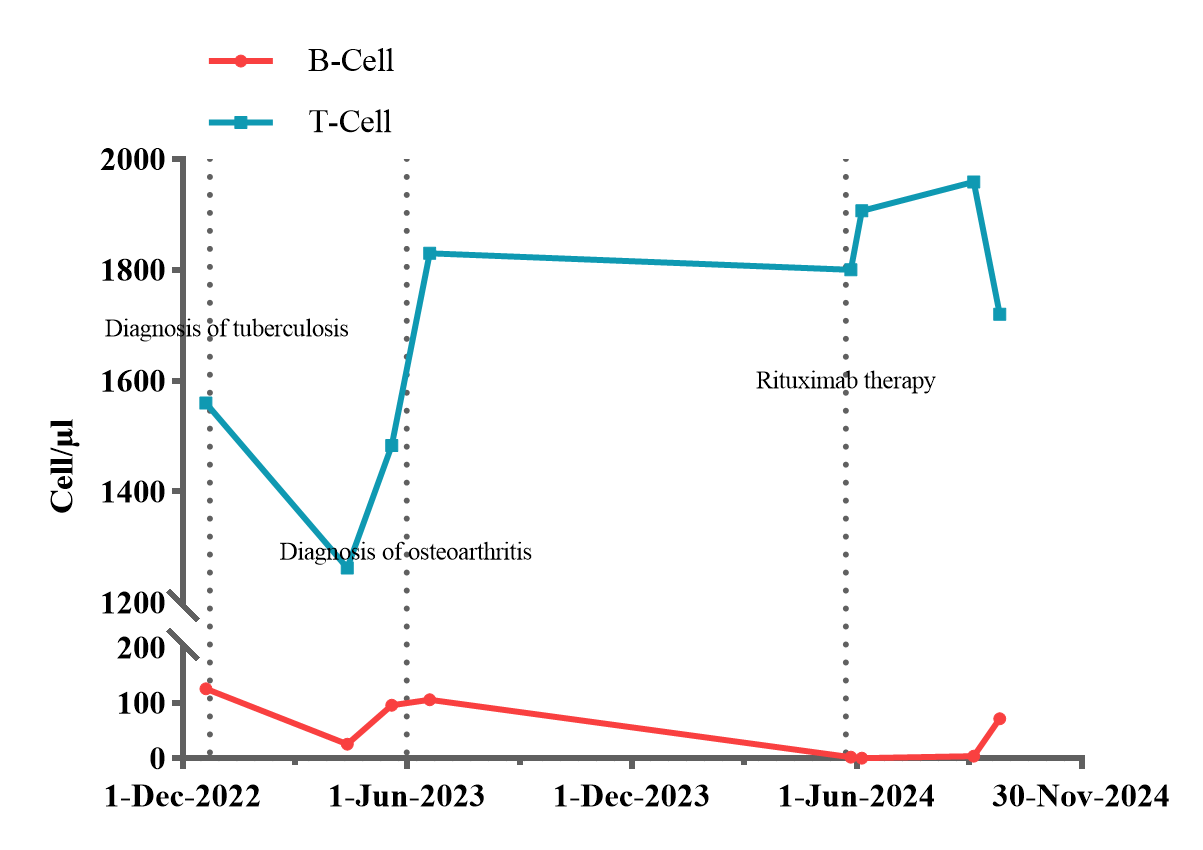


**Figure S4. The trend of changes in T-cell/B-cell counts throughout the course of the disease.**
